# SARS-CoV-2 excretion and genetic evolution in nasopharyngeal and stool samples from primary immunodeficiency and immunocompetent pediatric patients

**DOI:** 10.1186/s12985-025-02628-7

**Published:** 2025-01-13

**Authors:** Haifa Khemiri, Ilhem Ben Fraj, Alessio Lorusso, Najla Mekki, Iolanda Mangone, Mariem Gdoura, Adriano Di Pasqual, Cesare Cammà, Valeria Di Lollo, Asma Cherni, Henda Touzi, Amel Sadraoui, Zina Meddeb, Nahed Hogga, Imen Ben Mustapha, Mohamed-Ridha Barbouche, Monia Ouederni, Henda Triki, Sondes Haddad-Boubaker

**Affiliations:** 1 Laboratory of Clinical Virology, WHO Regional Reference Laboratory for Poliomyelitis and Measles for in the Eastern Mediterranean Region, Institut Pasteur de Tunis, University of Tunis El Manar, 13 place Pasteur, BP74 1002 le Belvédère, Tunis, Tunisia; 2 Research Laboratory “Viruses, Vectors and Hosts” (LR20IPT02), Institut Pasteur de Tunis, University of Tunis El Manar, Tunis, Tunisia; 3 Pediatric Department of the National Center of Bone Marrow Transplantation, Tunis, Tunisia; 4 Istituto Zooprofilattico Sperimentale dell’Abruzzo e del Molise, 64100 Teramo, Italy; 5 Laboratory of Transmission, Control and Immunobiology of Infection (LR11IPT02), Institut Pasteur de Tunis, University of Tunis El Manar, Tunis, Tunisia; 6 Faculty of Medicine, University of Tunis El Manar, Tunis, Tunisia; 7 Department of Microbiology, Immunology and Infectious Diseases, College of Medicine and Health Sciences, Arabian Gulf University, Manama, Bahrain

**Keywords:** Primary immunodeficiency, SNPs, Amino acid, Duration, COVID-19, Immunocompetent

## Abstract

**Background:**

Primary Immunodeficiency disorders (PID) can increase the risk of severe COVID-19 and prolonged infection. This study investigates the duration of SARS-CoV-2 excretion and the genetic evolution of the virus in pediatric PID patients as compared to immunocompetent (IC) patients.

**Materials and methods:**

A total of 40 nasopharyngeal and 24 stool samples were obtained from five PID and ten IC children. RNA detection was performed using RT-qPCR, and whole-genome sequencing was conducted with the NexSeq 1000 platform. Data analysis used the nextflow/viralrecon pipeline. Hotspot amino acid frequencies were investigated using GraphPad Prism v10. Phylodynamic analysis was conducted with BEAST software.

**Results:**

In IC children, the viral excretion period lasted up to 14 days in nasopharyngeal swabs, with an average duration of 7 days, and ranged from 7 to 14 days in stool samples. In PID patients, the viral RNA was detected in nasopharyngeal for periods between 7 and 28 days, with an average duration of 15 days, and up to 28 days in stool samples. Two SARS-CoV-2 variants were detected in PID patients: Delta (AY.122) and Omicron (BA.1.1). Patients with antibody and combined deficiencies, exhibited the most prolonged shedding periods in both nasopharyngeal and stool samples and one patient presented complications and fatal outcome. Specific Hotspot amino acid changes were detected in PID: A2821V and R550H (ORF1ab).

**Conclusion:**

Our findings underscore the prolonged excretion of SARS-CoV-2 RNA in patients with antibody and combined deficiencies. Thus, specialized care is essential for effectively managing PID patients.

**Supplementary Information:**

The online version contains supplementary material available at 10.1186/s12985-025-02628-7.

## Introduction

Coronavirus Disease 2019 (COVID-19) is an acute infectious respiratory illness caused by the Severe Acute Respiratory Syndrome Coronavirus 2 (SARS-CoV-2). It raised global concern since its emergence in 2019 [1]. SARS-CoV-2 is an enveloped single-stranded positive RNA virus. Its genome size, of approximately 30 kilobases (kb), includes 11 genes, that encode for 16 non-structural proteins, four structural proteins (spike protein (S), envelope protein (E), membrane protein (M) and nucleocapsid protein) and nine accessory proteins [2].

Primary immune deficiencies (PID), also known as inborn errors of immunity (IEI), are a group of genetic disorders affecting the immune system and resulting in increased susceptibility to various microorganism including viruses such as *Polioviruses* and *Enteroviruses* [3, 4]. Thus, the WHO Global Polio Eradication Initiative (GPEI) is, in part, based on poliovirus surveillance in PID patients [5, 6]. Similarly, PIDs are at heightened risk of prolonged SARS-CoV-2 excretion and severe COVID-19 manifestations [7]. The infection may last up to three months and patients typically require extended periods to fully clear the SARS-COV-2 [8, 9]. Furthermore, COVID-19 induces a significantly higher risk of mortality in immunodeficient (ID) patients, almost 150 times, greater than IC individuals [10].

Although COVID-19 has affected children less than adults, with generally more favorable outcomes, it is essential to highlight the increased vulnerability of ID children to COVID-19 [11, 12]. Within this population, a significant subset comprises individuals with PID involving more than 400 different ID types [13, 14]. The Centers for Disease Control and Prevention (CDC) [15] has identified PID as a condition that may increase the risk of severe COVID-19 and prolonged infection, a finding supported by various studies [3, 10, 14]. In addition to mild symptomology, they may exhibit severe symptoms such as drop of oxygen saturation, respiratory distress, fainting, tachypnea, pulmonary arrest [10, 16, 17]. Moreover, cardiological signs were reported such as cardiomegaly, severe cardiac enlargement, heart failure, requiring hospitalization and admission to an intensive care unit [10, 16, 17]. Given the prolonged excretion period, ID patients are at increased risk of developing severe symptoms and accumulating significant mutations [18]. This accumulation, particularly in the Spike gene, could accumulate the evolution and emergence of new SARS-CoV-2 lineages [19, 20]. The accumulated mutations can also enhance the virus’s ability to evade antibodies, facilitating escape from the host immune response and consequently promoting its spread within the community [20]. Consistently, it was suggested that the Omicron variant would have emerged in an immunocompromised patient after a prolonged period of viral replication [21]. Thus, it is crucial to track SARS-COV-2 evolution among immunodeficient patients and to investigate the potential effect of arising mutations for pathogenicity, viral escape and transmissibility. Furthermore, few studies focused on SARS-CoV-2 excretion by PID patients [22, 23]. To the best of our knowledge, only shedding in nasopharyngeal samples were described with loss of information about possible fecal excretion.

Tunisia, a country in the Middle East and North Africa (MENA) region, has a significant rate of consanguineous marriages within its population. This practice has led to a high prevalence of autosomal recessive forms of PID [24]. Nevertheless, in Tunisia, COVID-19 infection has been primarily studied in the general and pediatric populations, with limited interest for PID patients [25–30].

In this study, we described the SARS-COV-2 shedding in nasopharyngeal swabs and stool samples from PID pediatric patients in comparison with immunocompetent children. The genetic evolution and hotspot variable regions of excreted strains, during the period of shedding, were also investigated.

## Materials and methods

### Ethics statement

This study was approved by the Bio-Medical Ethics Committee of Pasteur Institute of Tunis, Tunisia (2020/14/I/LR16IPT/V1). It was performed under ethical standards according to the 1964 Declaration of Helsinki and its later amendments. Informed and written consent was obtained from the parents or legal tutors of children. All samples were investigated after de-identification with respect of patient anonymity.

### Patients

This study included fifteen patients, aged 7 months to 14 years, comprising ten immunocompetent (IC) children and five pediatric PID patients, all confirmed to have SARS-COV-2 infection. The inclusion criteria of IC children were the age of patients under 15 years old and the period of collected samples was aligned to the period of obtention of PID samples: 2021–2022. The IC group included four males and six females, aged between 7 months and 14 years (median = 11.5 years, average = 10.65 years). The PID group comprised four males and one female, aged between 10 months and 10 years (median = 5 years, average = 5.16 years). The PID patients were previously investigated at the Laboratory of Transmission, Control, and Immunobiology of Infections at Pasteur Institute of Tunis and followed at the Pediatric Department of the National Center of Bone Marrow Transplantation (Tunis, Tunisia). Immunological investigation includes phenotypic analysis using a combination of cell markers, serum immunoglobulin levels measurement, nitroblue tetrazolium test as well as proliferation assays.

All of patients have primary immunodeficiency (PID) characterized by antibody and combined deficiencies. Among them, one patient was diagnosed with X-linked inhibitor of apoptosis protein (XIAP) deficiency, another with ataxia-telangiectasia, two patients had agammaglobulinemia and one had hyper-IgM syndrome (Table 1). As described in Table 1, four patients received intravenous immunoglobulin (IGIV) and antibioprophylaxy. One patient underwent hematopoetic stem cell transplantation (HSCT) two months before contracting COVID-19 and received treatment with immunosuppressants, antivirals, antibiotics, and antifungal medications (Table 1).

**Table 1:** Characteristics of COVID-19 PID cases

Patients	P1	P2	P3	P4	P5
Age (years)	0.83	8	**7**	**5**	**5**
Gender	M	F	M	M	M
PID diagnosis	X-linked inhibitor of apoptosis protein (XIAP)	Ataxia Télangiectasia	Agamma-globulinemia	Agamma-globulinemia	Syndrome hyper IgM (HIGM)
Therapy*	HSCT (at 8 months)/ Valgancyclovir/ ciclosporine/ amoxicilline/ fluconazol/ cotrimoxazol	IGIV Cotrimoxazole	IGIV Cotrimoxazole Beclometasone	IGIV Cotrimoxazole	IGIV Cotrimoxazole
Number of nasopharyngeal samples	1	2	2	4	6
Number of stool samples	2	1	1	2	4
Shedding duration* (Days)	< 7	21	< 7	14	28
Comorbidities	CMV colitis	Bronchiectasis, myelodysplastic syndrome	Asthma, malformative uropathy	No	Psoriasis, bronchiectasis
COVID-19 manifestations	bloody diarrhea	Fever, cough, fatigue	Fever, cough	Fever, cough, headache	Fever, cough, rhinorrhorea, pneumonia
Outcome	Favorable	Favorable	Favorable	favorable	Transient improvement Hypoxemic pneumonia, hepatocellular insufficiency and multiple coinfections (*Hemophilus influenzae*, *Adenovirus*, *Enterovirus* and *Rhinovirus*) Died (5 months after COVID-19)
Lineage assignment	DELTA (AY.122.6)	DELTA (AY.122)	DELTA (AY.122)	OMICRON (BA.1.1)	OMICRON (BA.1.1)

Therapy*: usual cares

Shedding duration*: in nasopharyngeal swabs

**P**: patient, **HSCT**: Hematopoietic stem-cell transplantation, **IGIV**: Immune globulin intravenous, **CMV**: *Cytomegalovirus*

### Samples

A total of 40 nasopharyngeal and 24 stool samples were collected from all included patients. Fifteen nasopharyngeal swabs and ten stool samples were obtained from the five PID cases and 25 nasopharyngeal swabs and 14 stool samples were collected from the ten immunocompetent cases. Samples were gathered from February 2021 to February 2022. The PID patients were referred by The National Center of Bone Marrow Transplantation (CNGMO) to the Laboratory of Clinical Virology at the Pasteur Institute of Tunis (IPT), for SARS-COV-2 investigation. Samples obtained from immunocompetent children were collected by the Tunisian Ministry of Health staff, from home-quarantined individuals and received at Pasteur Institute. All participants underwent comprehensive monitoring, which included weekly collection of nasopharyngeal and stool samples until achieving negative SARS-COV-2 results.

### Virological investigation

#### Nucleic acid extraction and detection by RT-qPCR

Viral RNA was extracted from 140 µl of nasopharyngeal and stool samples supernatants after treatment according to the WHO guideline [31], using the QIAamp Viral RNA mini kit (Qiagen, Hilden, Germany), following the manufacturer instructions. The presence of SARS-CoV-2 RNA was determined with RT-qPCR using either HKU protocol [32] or NeoPlex™ COVID-19 Detection Kit (GeneMatrix, Seongnam, Kyonggi-do, South Korea) (https://www.fda.gov/media/138100/download), targeting RdRp and N genes [33].

#### Whole genome sequencing (WGS)

**Fig. 1:**
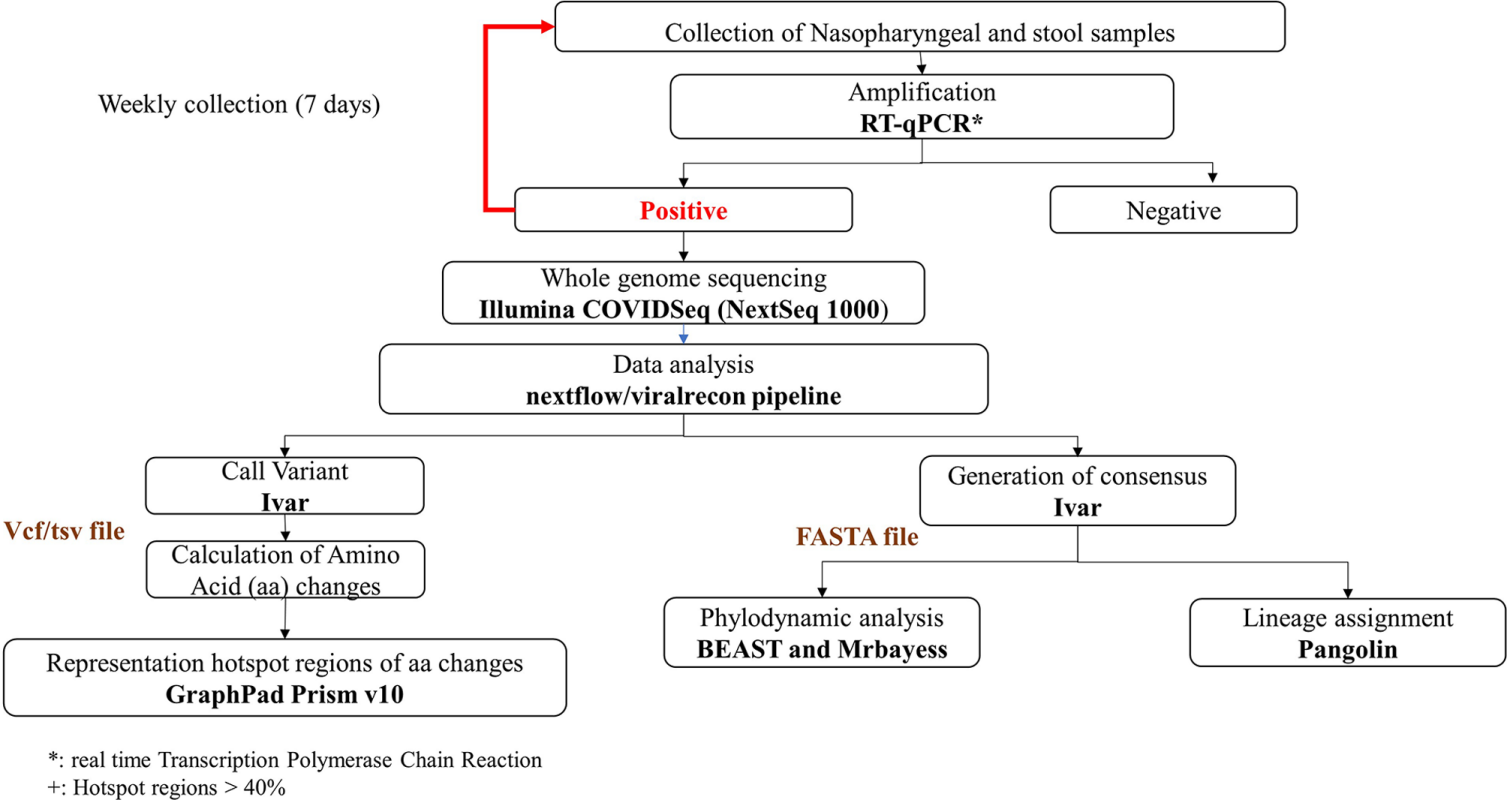
Workflow presenting the main methods used in this study. The output files colored in brown. The tools, software and techniques are presented in bold

Whole genome sequencing was conducted using of the Illumina COVIDSeq protocol [34] (Fig. 1). Library preparation was performed according to a validated protocol at the “National Reference Centre for Whole-Genome Sequencing of microbial pathogens at Istituto Zooprofilattico Sperimentale dell’Abruzzo e del Molise (IZSAM)”, using the Hamilton Microlab STAR Liquid Handling System (Hamilton Robotics, Reno, NV, USA) [27, 35]. Next Generation Sequencing (NGS) was accomplished with the NextSeq 1000 (Illumina Inc., San Diego, CA, USA), providing 150 bp paired-end reads.

### Generation of whole genome consensus sequence, variant calling, and identification

The data analysis was conducted using the nextflow/viralrecon pipeline version 1.0.0 [36] (Fig. 1). Quality control, and adapter trimming were executed with FastQC (version 0.11.9) and fastp (version 0.23.4), respectively. The reads were then aligned to the Wuhan-Hu-1 reference genome (MN908947.3) using bowtie2 (version 2.5.3) and samtools (version 1.15.1). Variant calling and consensus sequences were generated using Ivar tools (version 1.4.2). Subsquently, variant annotation was conducted using snpsift (version 4.3) and snpeff (version 5.0e). Then, quality control and visualization for raw reads, alignment, assembly, and variant calling results were carried out with MultiQC (version 1.20), which provides a single report for multiple samples. The identification of SARS-CoV-2 lineage and sub-lineage was achieved using Pangolin tools (version 4.3) via the web (https://pangolin.cog-uk.io/) [37]. The obtained sequences were submitted to the GISAID database (https://www.gisaid.org) [38] (Table S1).

### Hotspot analysis

Frequencies of amino acid changes were calculated and plotted based on their position on the Wuhan-Hu-1 reference genome, utilizing GraphPad Prism v10 (GraphPad Software, Inc., San Diego, California, United States) (Fig. 1). Common amino acid changes specific to the respective variants and sub-variants were excluded from the graph in the aim to highlight only the differences. Amino acid changes with a frequency greater than 40% were designated as hotspots.

### Phylodynamic analysis

A phylodynamic tree was built for patients who revealed prolonged virus excretion and provided more than three samples. Multiple alignment was performed using Clustal W available on MEGA Software (version 7.0.21). Two programs were used for phylodynamic tree construction: BEAST software (version 2.6.3) and MrBayes web server (version 3.2.6). The tree visualization was performed using Figtree software (version 1.4.3).

## Results

### Clinical presentation

The investigated children from both groups, exhibited predominantly mild COVID-19 form (80% PID, 70% IC). One PID patient presented severe form and one IC had moderate form (Table 2). In both groups, fever and cough were observed in 80% of the patients. In IC group, 60–80% of patients presented a runny nose, diarrhea, fatigue, and loss of smell and taste. Notably, in the PID group, bloody diarrhea and pneumonia were detected in 20% of the children (Table 2).

During their COVID-19 infection, four of PID patients received treatment, including azithromycin, salbutamol, paracetamol, and clarithromycin. No COVID complications were observed except in patient 5. Transient improvement of pneumonia was firstly observed and developed hypoxemic pneumonia, with hepatocellular insufficiency and lymphoproliferative syndrome. Multiple co-infections were diagnosed including *Enterovirus Echovirus 25*, *Rhinovirus*, *Haemophilus influenzae*, and *Adenovirus* and leading to death five months after COVID infection (Table 1).

**Table 2:** Clinical manifestations in primary immunodeficient (PID) and immunocompetent (IC) children in Tunisia

Clinical manifestations	PID Children (*n* = 5)	IC children (*n* = 10)
**Clinical Form**		
Mild	4 (80%)	7 (70%)
Moderate	0	1 (10%)
Severe	1 (20%)	2 (20%)
**Clinical features**		
Fever	4 (80%)	8 (80%)
Cough	4 (80%)	8 (80%)
Headache	1 (20%)	8 (80%)
Runny nose	0	1 (10%)
Loss of smell and taste	0	6 (60%)
Loss of appetite	0	1 (10%)
Urticaria	0	1 (10%)
Fatigue	0	7 (70%)
Body aches	0	7 (70%)
Bloody Diarrhea	1 (20%)	0
Diarrhea	0	8 (80%)
Vomiting	0	2 (20%)
Breathing difficulties	0	1 (10%)
Pneumonia	1 (20%)	0
**Lineage assignment**		
B.1.160	0	1 (10%)
Alpha (B.1.1.7)	0	2 (20%)
**Delta**	3 (60%)	2 (20%)
AY.122	2 (40%)	2 (20%)
AY.122.6	1 (20%)	0
**Omicron**	2 (40%)	5 (50%)
BA.1.1	2 (40%)	4 (40%)
BA.1.1.1	0	1 (10%)
**Outcome**		
Recovery	4 (80%)	10 (100%)
Death	1^*^	0

*: five months later

### Viral investigation

#### SARS-CoV-2 variant identification

Complete sequences of the SARS-CoV-2 genome were obtained from all cases in both studied populations. Four variants were detected: Alpha (B.1.1.7), B.1.160, Delta, and Omicron. All Delta and Omicron sequences belonged to sub-lineages AY.122 and BA.1.1, respectively (Table 2). In the PID patients were infected by AY.122 (3/5) and BA.1.1 (*n* = 2/5) variants and IC patients presented Alpha (*n* = 2), B.1.160 (*n* = 1), Delta (AY.122) (*n* = 2), and Omicron (BA.1.1) (*n* = 5) infections (Table 2).

#### Kinetics of SARS-COV-2 viral genome excretion

##### Kinetics in PID patients

**Fig. 2:**
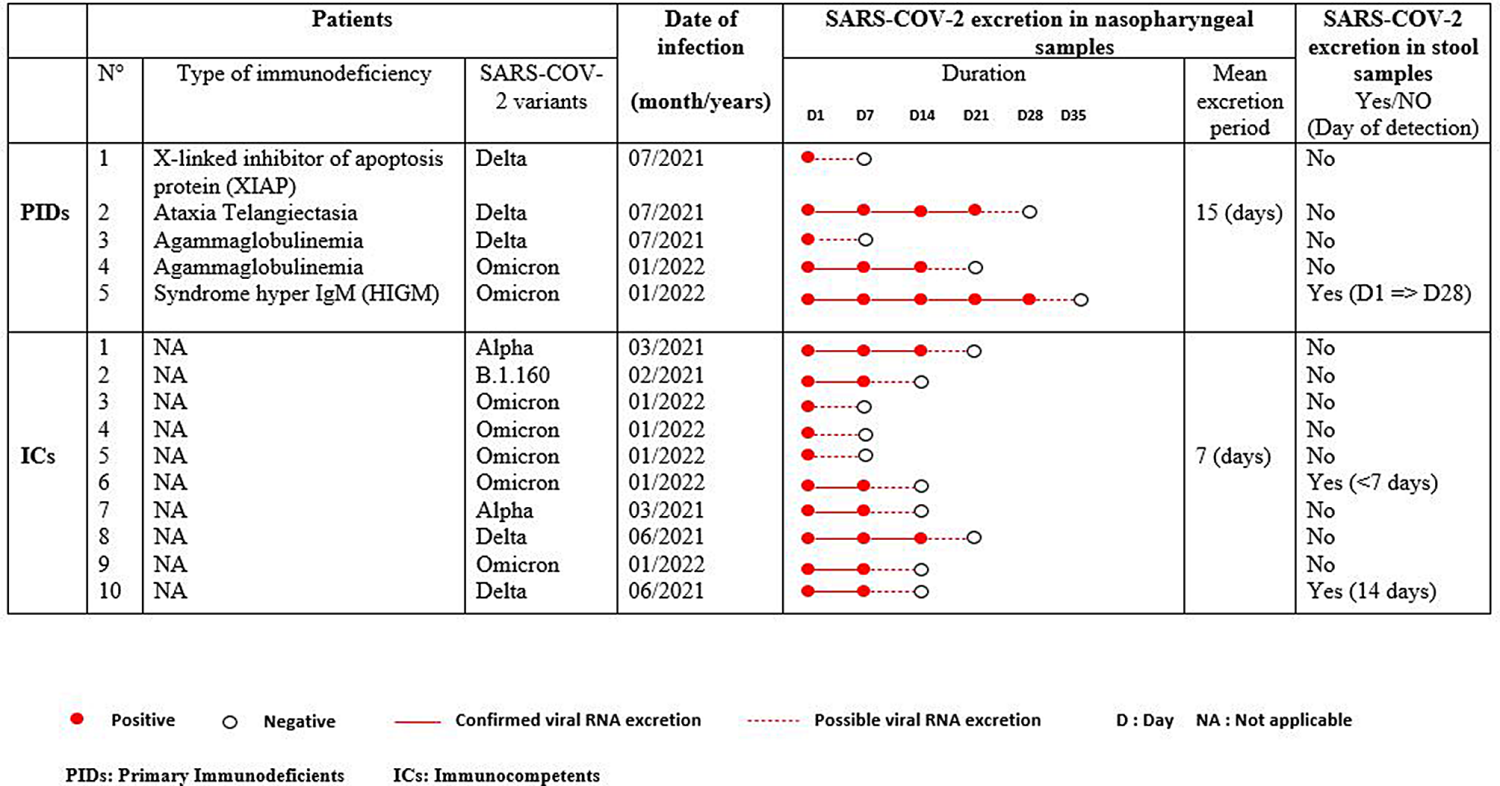
Kinetics of SARS-COV-2 viral genome excretion in nasopharyngeal and stool samples in Primary immunodeficiency (PID) and immunocompetent (IC) patients

Viral RNA was detected in 12 nasopharyngeal swabs obtained from the five PID patients. P1 and P3 excreted the virus for a period less than 7 days. P2, P4 and P5 continued to excrete SARS-CoV-2 genome for a period ranging between 14 and 28 days (Fig. 2). The mean duration of viral genome excretion in nasopharyngeal samples was 15 days. In contrast, only P5 showed viral RNA excretion in fecal sample which lasted for more than 28 days (Table 1). In day 35, nasopharyngeal and stool samples of P5 were negative for SARS-COV-2 Viral RNA (Fig. 2). Patient 5 showed the most prolonged excretion period in both nasopharyngeal and stool samples (Table 1, Fig. 2).

PID patients were infected by the Delta and Omicron variants. For the Delta variant, the viral RNA was detected for periods 7–21 days in nasopharyngeal samples. No excretion was reported in stool samples. For the Omicron variant, the viral RNA was detected for periods ranged between 14 and 28 days in nasopharyngeal samples and 28 days in stools.

##### Kinetics in IC patients

Viral RNA was detected in 21 nasopharyngeal swabs and 3 stool samples obtained from the ten and two IC patients, respectively. In nasopharyngeal samples, the shedding duration lasted up to 14 days in nasopharyngeal swabs, with an average duration of 7 days (Fig. 2). In stool samples, shedding duration ranged between 7 and 14 days.

According to SARS-COV-2 variants, for the Alpha, B.1.160 and Delta variants, the excretion period ranged between 7 and 14 days in nasopharyngeal swabs and stools. For the Omicron variant, the excretion period lasted up to seven days in nasopharyngeal swabs and less than seven days in stool sample (Fig. 2).

### Evolution of SNPs numbers

#### SNPs number in nasopharyngeal samples

Compared to the Wuhan reference sequence, the number of single nucleotide polymorphisms (SNPs) detected in sequences obtained from PID and IC children ranged from 25 to 54 and from 17 to 56, respectively. For the Delta variant, the number of SNPs varied from 25 to 41 and from 32 to 42 in PID and IC patients, respectively (Figure S1b-1d). Regarding the Omicron variant, the SNP count ranged from 42 to 54 and from 36 to 56 in the PID and IC groups, respectively (Figure S1a-1c). The number of SNPs varied from 17 to 33 in the Alpha variant sequences and from 26 to 27 in the B.1.160 variant sequences.

#### SNPs number in stool samples

The excretion in stool samples was detected in one PID patient (P5) and two IC patients (P6, P10). A total of five positive samples were obtained: two from the PID patient and three from the IC patients (Fig. 2). For the Omicron sequences, the number of SNPs varied from 14 to 52 in the two samples collected from PID patient (P5) and 47 in one sample obtained from the IC child. For the Delta sequences, the number of SNPs remains constant, equal to 42 in two successive samples obtained from IC patient.

### Amino acid frequency and hotspots

Hotspot amino acid changes were investigated in sequences obtained from nasopharyngeal samples, given the limited number of stool samples. The comparative analysis of amino acid changes concerned sequences obtained from same variants detected in PID and IC patients: Delta and Omicron. The common amino acid modifications of the Delta (sub-variant AY.122) and Omicron (sub-variant BA.1.1) variants comparing to the Wuhan sequence, were not considered in the graph in order to highlight only the differences between sequences obtained from immunodeficient and immunocompetent populations.

**Fig. 3:**
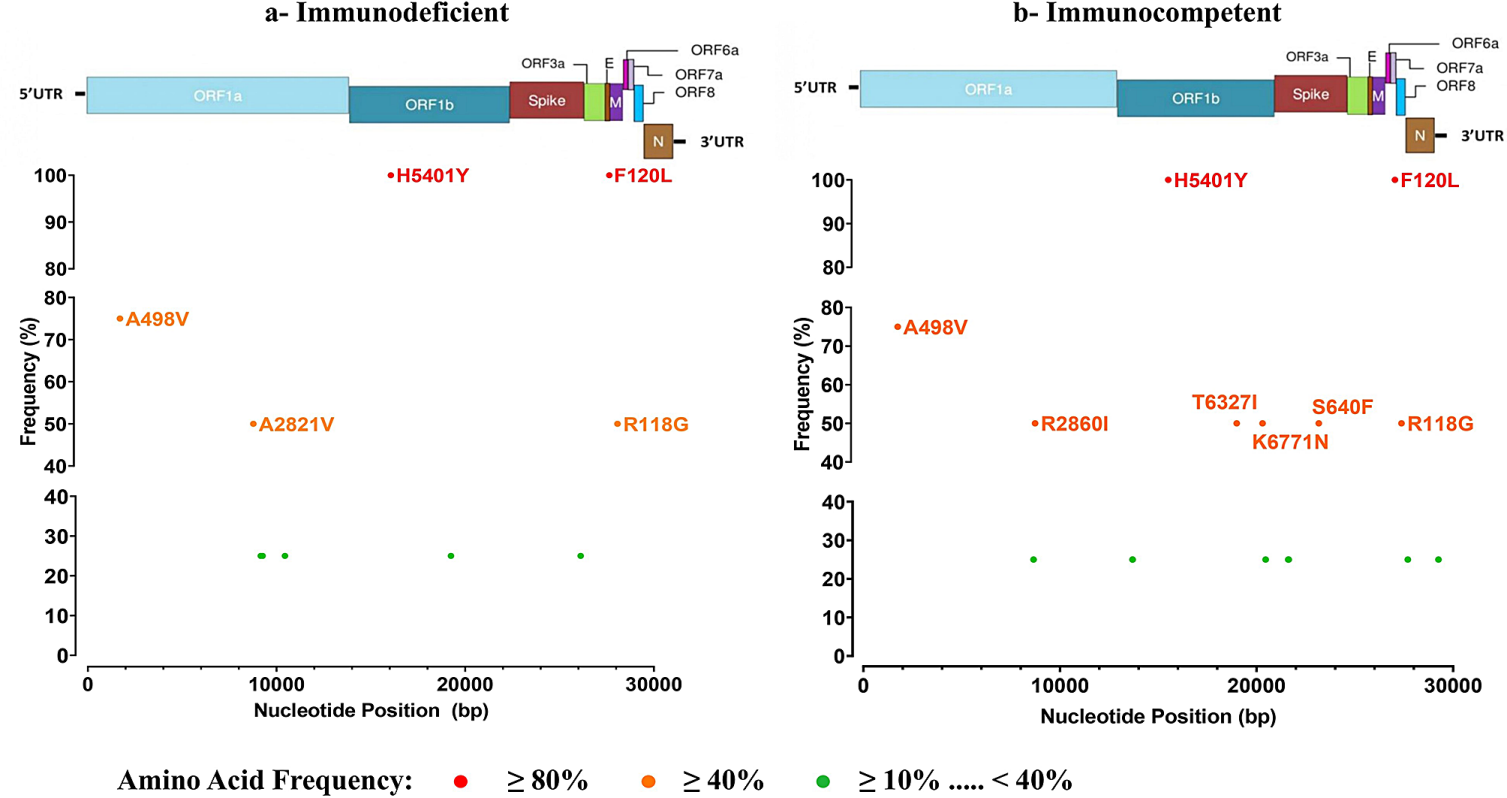
Frequency of amino acid changes presented in the Delta sequences obtained from Immunodeficient (**a**) and immunocompetent (**b**) patients. It was identified by referring to the reference sequence (Wuhan, NC_045512)

For the Delta variant, ten and 17 amino acid changes were detected in sequences obtained from PID and IC patients, respectively (Fig. 3a and b). In both groups, two amino acid changes were present in all sequences (100% frequencies): H5401Y (ORF1ab) and F120L (ORF8) and two others (A498V (ORF1ab) and R118G (ORF7a)) were present with frequencies of 75% and 50%, respectively. In PID patients, A2821V (ORF1ab) was detected in 50% of sequences. In IC children, four other amino acid changes were detected: R2860I (ORF1ab), T6327I (ORF1ab), K6771N (ORF1ab), S640F (S), each with a frequency of 50%.

**Fig. 4:**
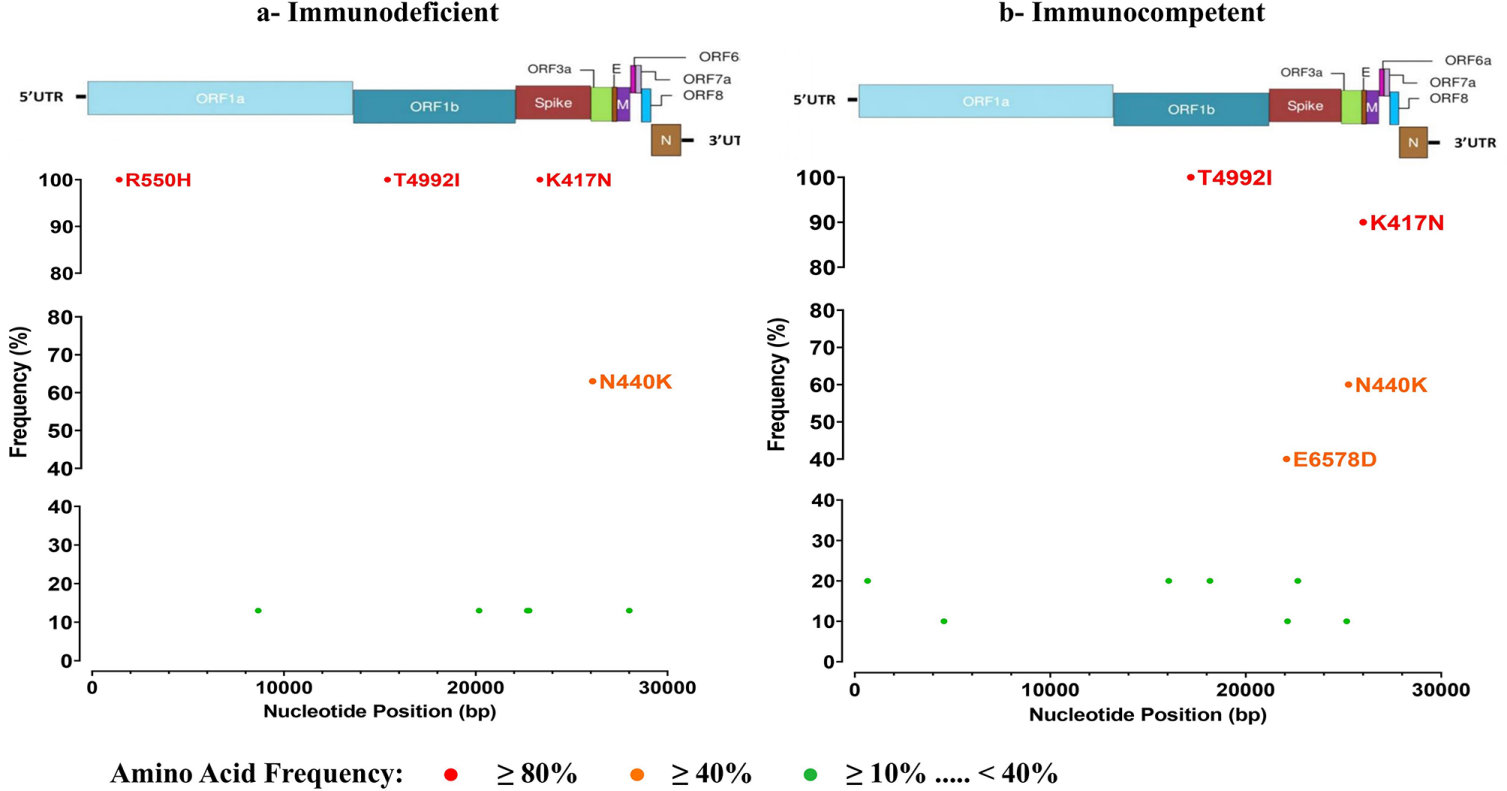
Frequency of amino acid changes presented in Omicron (BA.1.1 sub-variant) sequences obtained from immunodeficient (**a**) and immunocompetent (**b**) patients. It was identified by referring to the reference sequence (Wuhan, NC_045512)

For the Omicron variant (BA.1.1 sub-variant), ten and eleven amino acid changes were detected in sequences obtained from PID and IC children, respectively (Fig. 4a and b). In both groups, three amino acid changes were detected: T4992I (ORF1ab) and K417N (S) in all sequences (100% frequencies) and N440K (S) with 63% and 60% frequencies in PID and IC sequences, respectively. For the PID sequences, R550H (ORF1ab) was detected in all sequences (100 frequencies). For IC children, E6578D (S) was detected in 40% of sequences.

### Phylodynamic analysis

**Fig. 5:**
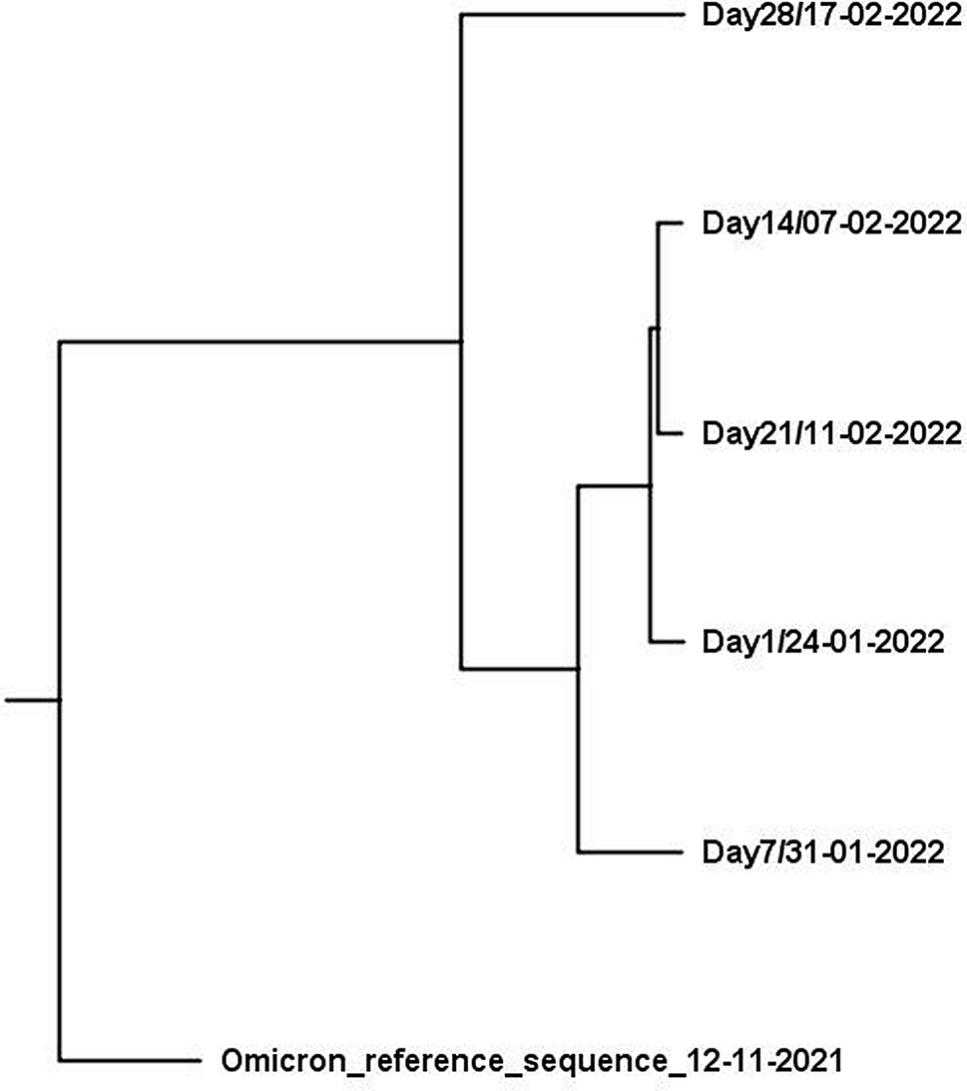
Phylogenetic tree obtained with BEAST and visualized with Figtree

The phylodynamic tree was constructed for sequences obtained from the patient 5 who exhibited prolonged virus excretion and provided more than three samples. It was generated using both BEAST and MrBayes softwares. As showin in Fig. 5, Beast generated tree revealed close genetic relationship between sequences obtained on days 1, 7, 14, and 21, while the sample collected on day 28 exhibits considerable genetic distance from the preceding sequences (Fig. 5). Similar clustering was obtained by MrBayes analysis (Figure S2).

## Discussion

Patients with primary immunodeficiency diseases (PID) are specifically recognized as being at higher risk of severe COVID-19 outcomes [1, 15]. Few studies have specifically addressed COVID-19 infection in pediatric PID cases. The majority of studies have described COVID-19 in other types of immunodeficiency, such as cancer [39], liver transplantation [40], B-cell acute lymphoblastic leukemia [17], non-Hodgkin Lymphoma [41], chronic lymphocytic leukemia [42] and HIV [43]. In Tunisia, a country in the MENA region with a high frequency of consanguinity, there is a considerable rate of PID [22]. However, COVID-19 infection was only investigated in the general and pediatric Tunisian populations with a limited interest on PID patients [23–30].

In this study, five PID children were followed during their SARS-COV-2 infection in comparison with ten IC children. The patient with XIAP deficiency underwent hematopoietic stem cell transplantation (HSCT) two months before contracting COVID-19. As documented, recipients of stem cell transplants can remain severely immunocompromised up to twelve months post-transplantation, particularly if they are still under immunosuppressive medications and restoration of T-cell and B-cell immunity [44], which was the case of this patient. Among PID patients, three presented antibody deficiencies: agammaglobulinemia in Patient3 and Patient4, and hyper IgM syndrome in Patient5. Patients with agammaglobulinemia experienced mild COVID-19 symptoms, whereas the patient with hyper IgM syndrome exhibited a severe form of the disease, including pneumonia and *Enterovirus* co-infection. He died five months after multiple post-COVID-19 complications such as hepatocellular failure, and multiple co-infections: *Haemophilus influenzae*, *adenovirus*, *enterovirus*, *and rhinovirus*. According to Delavari et al. (2021), disease severity may be higher in children with combined immunodeficiencies, antibody deficiencies, and immune dysregulation, compared to those with other types of immunodeficiency [10]. These children are generally hospitalized and may develop severe respiratory symptoms including distress, tachypnea as well as cardiac and pulmonary arrest [10]. It was also reported that patients with antibody deficiencies are the predominant group among “inborn errors of immunity” affected by COVID-19 [16]. The same PID case, with hyper IgM syndrome, exhibited prolonged shedding in both nasopharyngeal and stool samples for 28 days. Other PID cases (P2, P4), presenting antibodies and combined deficiencies, presented excretion periods up to 21 and 14 days in nasopharyngeal samples only. For the IC group, two patients presented excretion, up to 14 days and all patients recovered without any complications. According to the Centers for Disease Control and Prevention, immunocompromised patients may have persistent viral shedding beyond 20 days [45]. Moreover, other studies have revealed that the duration of viral shedding in both nasopharyngeal (NP) and fecal samples can extend from 42 days to 3 months in PIDs [1, 8, 9]. Our study provides another testimony on the prolonged shedding and severe outcome of antibodies and combined deficiencies patients when infected by SARS-CoV-2. The prolonged SARS-CoV-2 excretion may be linked to significant impairment of adaptive immunity. For instance, Calvet J and colleagues found that critically ill SARS-CoV-2 patients exhibited a decrease in CD3 + CD4 + T cells compared to non-critical patients [46].

Regarding the number of SNPs detected in investigated sequences, limited information within the PID population were provided in the literature. Most studies have documented SARS-CoV-2 mutations in other types of immunodeficiency [19, 41, 47].

Our study showed similar results for both PID and IC patients varying from 25 to 42 in the Delta variant and from 36 to 56 in the Omicron variant. This finding highlights the higher number of SNPs observed in Omicron variants across both groups. Similarly, other studies have reported that the Omicron variant exhibits a greater number of mutations compared to other SARS-CoV-2 variants [48–50]. These mutations are distributed across different regions of the viral genome, with a high number of mutations in the Spike protein. The high mutational load of the Omicron variant contributes to its increased transmissibility, reduced susceptibility to neutralizing antibodies, and ability to partially evade vaccine-induced immunity.

Furthermore, the phylodynamic analysis of sequences obtained from PID patient P5 demonstrated a close genetic relationship among viral sequences collected from day 1 to day 21 followed by an increased diversity from day 21 to day 28 of infection. This divergence is likely attributed to the host’s inability to mount an effective immune response due to the immunodeficiency conditions that lead to the accumulation of multiple mutations over time. Prolonged infections, especially in patients with primary immunodeficiencies (PIDs), provide an extended opportunity for the virus to adapt and evolve under compromised host-specific conditions [51]. These conditions may favor the emergence of mutations and enhance viral fitness, allowing immune evasion and even improve the virus’s transmissibility or pathogenicity [19].

For advanced investigation of genomic sequences, the frequencies of mutations and amino acid changes in the Delta and Omicron variants were calculated in both patient groups. Interestingly, amino acid changes A2821V (ORF1ab) and R550H (ORF1ab) were detected only in PID patient. Both changes were not well documented and deserve further investigation.

Other amino acid changes were detected in both populations with significant frequencies (50–100%): H5401Y (ORF1ab), F120L (ORF8), T4992I (ORF1ab), K417N (S), A498V (ORF1ab), R118G (ORF7a) and N440K (S). The majority of these substitutions have been previously documented in the literature. The amino acid changes K417N and N440K have been shown to enhance viral transmissibility and infectivity, potentially contributing to immune evasion [52, 53]. They play a crucial role in impacting the virus’s ability to bind to the ACE2 receptor by stabilizing the RBD–ACE2 complex [54]. The amino acid changes, H5401Y and A498V, were detected in Delta variant sequences [54, 55], with A498V being particularly prevalent in Tunisian Delta sequences [27]. These mutations are located in the ORF1ab region and may influence the function of the non-structural protein 3, which plays a critical role in viral processes such as replication, pathogenicity, and interaction with the host immune system [55].

In the other hand, the amino acid changes, F120L in ORF8 and R118G in ORF7a seem adversely affecting the viral replication and the virus’s interaction with the host immune system [56, 57]. In contrast, specific details about the T4992I (ORF1ab) change were not well documented.

Several other amino acid changes (R2860I, T6327I, K6771N, S640F, E6578D) have been identified exclusively in sequences from IC patients; the exact roles of these mutations were not identified [58].

Overall, the obtained results, PID’s type such as combined and antibody immunodeficiencies, prolonged excretion as well as significant genetic diversity may guide treatment decisions for immunocompromised children with COVID-19 [3, 16, 59].

Immunomodulatory therapies, including dexamethasone and inhibitors targeting interleukin-6 or Janus kinase, are particularly effective in treating severe or critical cases of COVID-19. In less severe cases, antiviral treatments, such as remdesivir and nirmatrelvir-ritonavir, have shown efficacy. However, anti-SARS-CoV-2 monoclonal antibodies are no longer recommended due to the emergence of variants resistant to these therapies [60]. Despite these advancements, there is a lack of robust data to determine whether these treatments are effective in reducing viral persistence in children with PIDs.

It is worthy to note that this study included a limited number of samples from children with PID. Indeed, it was challenging to follow PID children and collecting successive nasopharyngeal and stool samples. Such type of finding is very rare. For this reason, our study provides valuable insights into the prolonged viral shedding and clinical outcomes of this type of vulnerable patients. Future investigations, involving a larger PID patients at an international scale should be considered to support these findings and further elucidate the mechanisms underlying viral shedding in this at-risk group as well as defining treatment strategies for PID management [61].

## Conclusion

In summary, patients with antibody and combined immunodeficiencies excreted viral RNA for longer periods compared to immunocompetent individuals in both nasopharyngeal and stool samples. Post-COVID-19 complications was also notified in one patient leading to fatal outcome. Extended genomic monitoring allowed detection of significant genetic divergence in the final sequence, likely due to the accumulation of multiple mutations over time. These findings emphasize the importance of long-term surveillance of viral evolution in PID patients. Such monitoring not only helps in understanding the dynamics of within-host viral evolution but also contribute to a better management of health conditions and may guide treatment decision for immunocompromised patients with COVID-19 infection.

## Electronic supplementary material

Below is the link to the electronic supplementary material.

Supplementary Material 1: Additional file 1: Table S1: Accession ID and virus name of complete genome SARS-CoV-2 sequences generated in this study and submitted in GISAID

Supplementary Material 2: Additional file 2: Figure S1: Variation of the number of SNPs in nasopharyngeal samples over time in immunodeficient (a-b) and immunocompetent (c-d) patients in Omicron (a-c) and Delta (b-d) variants

Supplementary Material 3: Additional file 3: Figure S2: Phylogenetic tree obtained with MrBayes

## Data Availability

The sequences generated in this work are available at GISAID database (https://www.gisaid.org) under the following accession numbers: EPI_ISL_19265883, EPI_ISL_19265884, EPI_ISL_19265885, EPI_ISL_19265886, EPI_ISL_19265887, EPI_ISL_19265888, EPI_ISL_19265889, EPI_ISL_19265890, EPI_ISL_19265891, EPI_ISL_19265892, EPI_ISL_19265893, EPI_ISL_19265894, EPI_ISL_19265895, EPI_ISL_19265896, EPI_ISL_19265897, EPI_ISL_19265898, EPI_ISL_19265899, EPI_ISL_19265900, EPI_ISL_19265901, EPI_ISL_19265902, EPI_ISL_19265903.

## Abbreviations

PID: Primary Immunodeficiency
COVID-19: Coronavirus Disease 2019
IC: Immunocompetent
SARS-CoV-2: Severe Acute Respiratory Syndrome Coronavirus 2
RT-qPCR: Real-Time Quantitative Reverse Transcription polymerase chain reaction
BEAST: Bayesian Evolutionary Analysis Sampling Trees
RNA: Ribonucleic acid
ORF: Open Reading Frame
IEI: Inborn Errors of Immunity
ID: Immunodeficient
CDC: Centers for Disease Control and Prevention
MENA: Middle East and North Africa
XIAP: X-linked inhibitor of apoptosis protein
IGIV: Intravenous immunoglobulin
HSCT: Hematopoietic stem cell transplantation
HKU: Hong Kong University
WGS: Whole Genome Sequencing
NGS: Next Generation Sequencing
SNPs: Single Nucleotide Polymorphisms
S: Spike
HIV: Human Immunodeficiency Virus
NP: Nasopharyngeal
ACE2: Angiotensin-Converting Enzyme 2
RBD: Receptor-Binding Domain
