# Supplementary material for: SARS-CoV-2 excretion and genetic evolution in nasopharyngeal and stool samples from primary immunodeficiency and immunocompetent pediatric patients
Source: Virol J. 2025 Jan 13;22:9. doi: 10.1186/s12985-025-02628-7 (PMC11730810; doi:10.1186/s12985-025-02628-7)
Supplement: Supplementary file 1 — Supplementary Material 1: Additional file 1: Table S1: Accession ID and virus name of complete genome SARS-CoV-2 sequences generated in this study and submitted in GISAID [file 12985_2025_2628_MOESM1_ESM.docx]

| **Accession ID** | **Virus name** |
| --- | --- |
| EPI_ISL_19265883 | hCoV-19/Tunisia/Tunis-S431/2021 |
| EPI_ISL_19265884 | hCoV-19/Tunisia/Tunis-S414/2021 |
| EPI_ISL_19265885 | hCoV-19/Tunisia/Tunis-S023/2022 |
| EPI_ISL_19265886 | hCoV-19/Tunisia/Tunis-A136/2021 |
| EPI_ISL_19265887 | hCoV-19/Tunisia/Tunis-A132/2021 |
| EPI_ISL_19265888 | hCoV-19/Tunisia/Tunis-A043/2022 |
| EPI_ISL_19265889 | hCoV-19/Tunisia/Tunis-A037/2022 |
| EPI_ISL_19265890 | hCoV-19/Tunisia/Tunis-A031/2022 |
| EPI_ISL_19265891 | hCoV-19/Tunisia/Tunis-A026/2021 |
| EPI_ISL_19265892 | hCoV-19/Tunisia/Tunis-A019/2022 |
| EPI_ISL_19265893 | hCoV-19/Tunisia/Tunis-A018/2022 |
| EPI_ISL_19265894 | hCoV-19/Tunisia/Tunis-A012/2022 |
| EPI_ISL_19265895 | hCoV-19/Tunisia/Tunis-8138/2021 |
| EPI_ISL_19265896 | hCoV-19/Tunisia/Bizerte-A210/2021 |
| EPI_ISL_19265897 | hCoV-19/Tunisia/Ben-Arous-A021/2022 |
| EPI_ISL_19265898 | hCoV-19/Tunisia/Ben-Arous-9382/2021 |
| EPI_ISL_19265899 | hCoV-19/Tunisia/Beja-S036/2022 |
| EPI_ISL_19265900 | hCoV-19/Tunisia/Beja-A052/2022 |
| EPI_ISL_19265901 | hCoV-19/Tunisia/Beja-A050/2022 |
| EPI_ISL_19265902 | hCoV-19/Tunisia/Beja-A046/2022 |
| EPI_ISL_19265903 | hCoV-19/Tunisia/Beja-A041/2022 |

**Table S1:** Accession ID and virus name of complete genome SARS-CoV-2 sequences generated in this study and submitted in GISAID.
