# Supplementary figures and images for: SARS-CoV-2 excretion and genetic evolution in nasopharyngeal and stool samples from primary immunodeficiency and immunocompetent pediatric patients
Source: Virol J. 2025 Jan 13;22:9. doi: 10.1186/s12985-025-02628-7 (PMC11730810; doi:10.1186/s12985-025-02628-7)

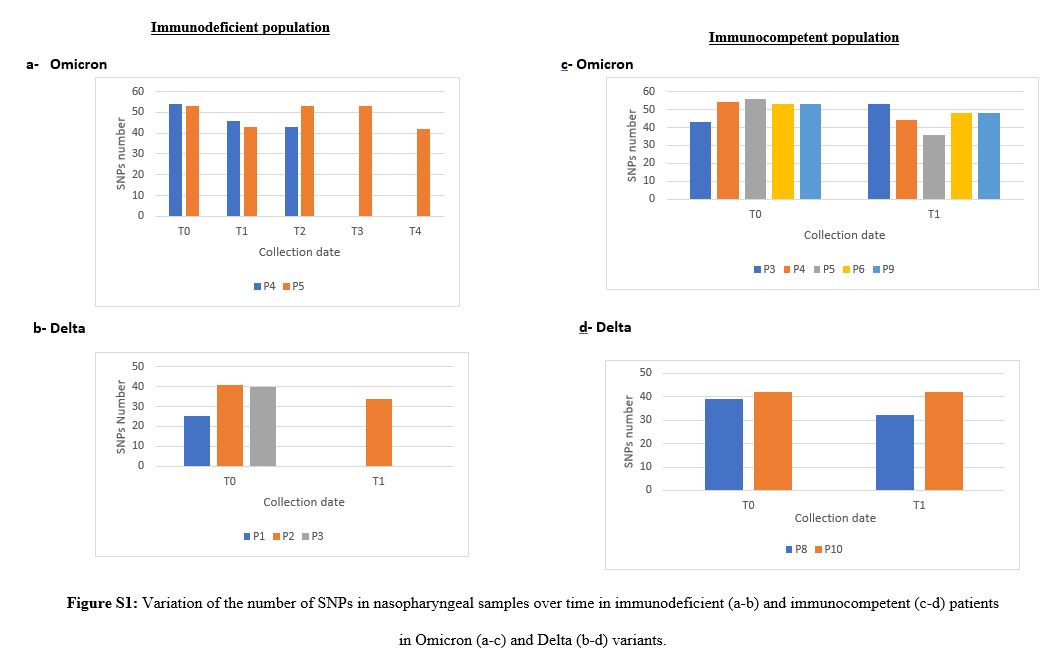

Supplement: Supplementary file 2 — Supplementary Material 2: Additional file 2: Figure S1: Variation of the number of SNPs in nasopharyngeal samples over time in immunodeficient (a-b) and immunocompetent (c-d) patients in Omicron (a-c) and Delta (b-d) variants [file 12985_2025_2628_MOESM2_ESM.jpg]

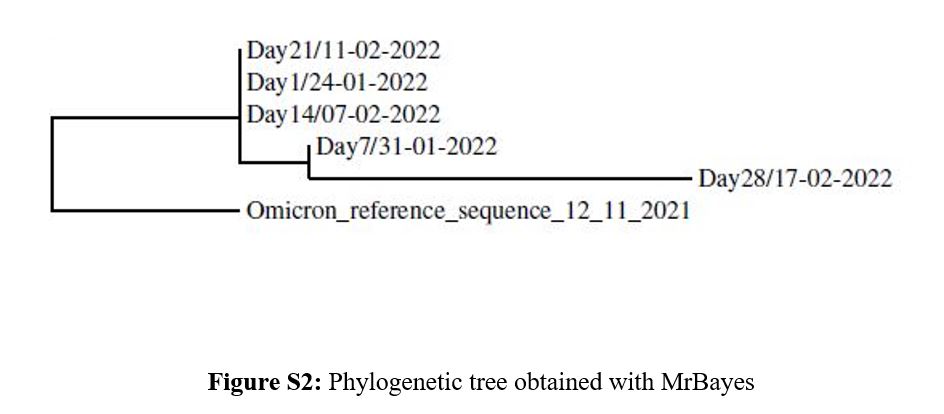

Supplement: Supplementary file 3 — Supplementary Material 3: Additional file 3: Figure S2: Phylogenetic tree obtained with MrBayes [file 12985_2025_2628_MOESM3_ESM.jpg]
